# Membranoproliferative Glomerulonephritis Associated with Nivolumab Therapy

**DOI:** 10.1155/2020/2638283

**Published:** 2020-02-24

**Authors:** Jessica Cruz-Whitley, Nolan Giehl, Kuang-Yu Jen, Brian Young

**Affiliations:** ^1^Department of Internal Medicine, University of California at Davis, Sacramento, CA, USA; ^2^Department of Pathology, University of California at Davis, Sacramento, CA, USA; ^3^Department of Internal Medicine, Division of Nephrology, University of California at Davis, Sacramento, CA, USA

## Abstract

Nivolumab is an immune checkpoint inhibitor that targets programmed death-1 on T cells and is designed to amplify an immunologic reaction against cancer cells. However, upregulation of the immune system with checkpoint inhibition is nonspecific, and it can be associated with certain renal side effects, the best documented of which is acute tubulointerstitial nephritis. We present a unique case of a patient with acute kidney injury associated with nephrotic syndrome shortly after starting nivolumab therapy for metastatic anal carcinoma. Subsequent renal biopsy revealed membranoproliferative glomerulonephritis (MPGN). We believe this represents the first reported direct case of nivolumab-associated MPGN. As immunotherapy becomes more widely used in cancer treatment, particular attention must be paid to possible consequences of immune checkpoint inhibitors.

## 1. Introduction

A common survival strategy employed by cancer cells is to activate immune checkpoints in order to evade immune surveillance. A new class of drugs known as immune checkpoint inhibitors counteracts this process, reactivating immunologic recognition and destruction of tumor cells. [1, 2] These drugs have revolutionized the field of oncology and are increasingly used to treat various types of malignancies.

Nivolumab is one of these immunotherapy agents and functions as an antagonistic antibody against the immune checkpoint cell surface receptor programmed death-1 (PD-1). Autoimmunity may be a consequence of immunotherapy, though renal side effects are fairly infrequent (1%) and when present are most often related to acute tubulointerstitial nephritis (AIN).[3, 4] Nephrotic syndrome as the major presenting adverse effect related to immunotherapy is exceedingly rare, with cases of minimal change disease and focal segmental glomerulosclerosis only recently being reported. [5–8] With more experience and widespread use of immune checkpoint inhibitors, there appears to be increasing recognition that a broader spectrum of immune-related kidney injury may be experienced.

In this report, we present a patient who developed acute kidney injury and nephrotic syndrome shortly after starting nivolumab for anal carcinoma. Renal biopsy revealed features of membranoproliferative glomerulonephritis (MPGN). Given that her renal clinical course closely followed the timing of nivolumab initiation and subsequent discontinuation, her MPGN was thought to be most likely due to nivolumab exposure. To our knowledge, this is the first reported case of MPGN directly related to nivolumab therapy.

## 2. Case Report

### 2.1. Clinical History and Initial Laboratory Data

A 75-year-old female with a history of non-Hodgkin lymphoma, status-post allogenic bone marrow transplant 10 years ago, and heart failure from restrictive cardiomyopathy was diagnosed with metastatic squamous cell anal carcinoma. She underwent laparoscopic loop diverting colostomy with combined 5-fluororuacil and mitomycin C with radiation. Recurrence was noted several months after initial therapy. She was then treated with nivolumab (2.4 mg/kg) monotherapy. The patient had relatively preserved renal function with the serum creatinine (SCr) level of 1.07 mg/dL prior to starting nivolumab. Over the course of five cycles of nivolumab received over a two month period, she developed acute kidney injury with SCr rise that peaked at 1.63 mg/dL. She had no other identifiable nephrotoxic exposures or medications at that time. Nivolumab was held, and she was empirically started on prednisone 40 mg daily by her oncologist for presumed acute tubulointerstitial nephritis.

Physical exam revealed bilateral lower extremity edema and an ostomy bag. The patient had chronic anemia and thrombocytopenia, as well as variable hypoalbuminemia (as low as 2.6 g/dL). Urine dipstick was notable for proteinuria and hematuria, but without significant pyuria. She had an estimated 12.7 grams of protein on spot urine protein-creatinine ratio (UPCR). Urine microscopy showed <15 nondysmorphic red cells per high power field. Workup included antinuclear, antidouble-stranded DNA, proteinase 3, and myeloperoxidase antibodies, which were all negative. C3 and C4 were low at 76 mg/dL and 10 mg/dL, respectively. There was no evidence for monoclonal gammopathy, and tests for active hepatitis B and C were negative. Kidney ultrasound was overall unremarkable. After four weeks of prednisone, her kidney function was largely unchanged.

### 2.2. Renal Biopsy Findings

**Figure 1:**
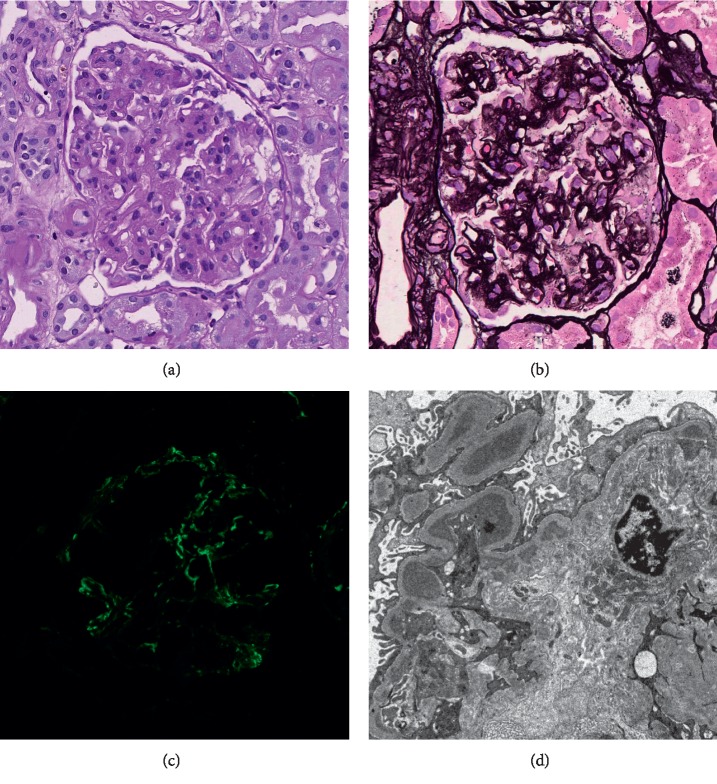
Kidney biopsy findings. (a) Periodic acid-Schiff and (b) Jones methenamine silver-stained sections demonstrate mild-to-moderate mesangial widening and minimal endocapillary proliferation with associated segmental double contours of the glomerular basement membrane. (c) C3-dominant staining is present in the mesangium and peripheral glomerular capillary loops. (d) Electron microscopy reveals subendothelial immune deposits with evidence of glomerular basement membrane reduplication.

To assess the cause of the patient's persistent proteinuria, a kidney biopsy was performed. Light microscopy revealed mild to moderate mesangial widening, mild mesangial hypercellularity, and minimal endocapillary proliferation (Figure 1(a)). Segmental double contours of the glomerular basement membrane were identified, but no large subendothelial deposits or hyaline thrombi were seen (Figure 1(b)). Mild, patchy interstitial inflammation consisting of mononuclear leukocytes was present along with acute tubular injury, but the degree of interstitial inflammation was relatively trivial. On immunofluorescence microscopy, there was C3-dominant staining (2+) with less-intense IgM staining (1+) in the mesangium and scattered along the glomerular capillary loops (Figure 1(c)). Ultrastructural analysis revealed scattered subendothelial and mesangial electron-dense immune deposits with associated glomerular basement membrane reduplication and mesangial cell interposition (Figure 1(d)). The majority of the podocyte foot processes were effaced. The combination of these findings in the context of the patient's laboratory values supported the diagnosis of MPGN.

### 2.3. Treatment and Clinical Course

Due persistent acute kidney injury and nephrotic syndrome, nivolumab was held for approximately eight weeks, while prednisone was continued. Her course was complicated by Klebsiella urosepsis requiring a rapid tapering of her prednisone. As the next treatment course was being discussed, she was admitted once more to the hospital with altered mental status and hypercalcemia, at which point her cancer was discovered to be progressive. She ultimately chose to be discharged on hospice, passing away shortly after. Around that time, SCr was 1.17 mg/dl, and spot UPCR was 1.1 g/g, both approximately 4 months after her kidney biopsy.

## 3. Discussion

Immunotherapy is a revolutionary approach to cancer management, particularly in advanced malignancies that have progressed despite traditional chemotherapy. One approach targets the interaction between programmed cell death-1 (PD-1), a transmembrane receptor protein expressed on immune cells and its ligand (PD-L1). PD-L1 is found on many native cell types, and induces self-tolerance by preventing T-cell-mediated apoptosis and downregulating T-effector cell activity. Cancer cells can take advantage of these checkpoint mechanisms by also expressing PD-L1, thus avoiding detection by the host immune system. Novel checkpoint inhibitors such as nivolumab, an anti-PD-1 monoclonal antibody, facilitate host immune response to cancer cells by eliminating tumor cell PD-L1 binding to PD-1 on T cells. [1].

Immune-related adverse events can occur as a consequence of checkpoint inhibitor due to impaired self-tolerance from the loss of T-cell inhibition. Adverse events are common in several large organ systems, including colitis, pneumonitis, hepatitis, skin toxicities, and endocrinopathies. [9] Kidney adverse effects are rare, accounting for approximately 1% of all immune-related adverse events. [10] AIN is the most common kidney pathology associated with checkpoint inhibitors, including nivolumab. [3–5] In clinical trials of nivolumab, kidney dysfunction occurred in 1.2% of patients receiving nivolumab as a single agent with a median time to onset of 4.6 months. [11] In case reports, the onset to acute kidney injury (AKI) from AIN varied from 21 days to 18 months. Discontinuation of the drug was the mainstay of treatment, followed by prednisone administration in most cases with partial to full recovery of renal function. [2].

Our patient had AKI approximately 2 months into her nivolumab treatment. Atypical for AIN, the patient had nephrotic syndrome with proteinuria estimated by the spot ratio of over 12 grams at the time of consultation. Although there was no formal assessment of proteinuria prior to her nephrology visit, urine dipsticks done the year before were only significant for trace to 1+ proteinuria, suggesting the nephrotic syndrome was of recent and rapid onset. Because of this unusual finding, the patient underwent kidney biopsy.

The kidney biopsy showed MPGN as the primary cause of her AKI and nephrotic syndrome. Nivolumab has only previously been associated with nephrotic syndrome from focal segmental glomerulosclerosis. [6–8] Nephrotic syndrome from minimal change disease has also been reported with other checkpoint inhibitors such as pembrolizumab, another PD-1 antibody, and ipilimumab, an anti-CTLA4 antibody. Other patterns of glomerular disease have been documented with checkpoint inhibitors, including IgA dominant glomerulonephritis and lupus-like glomerulonephritis. [12–15] The mechanism by which nivolumab may cause glomerular disease remains to be elucidated. It is possible that loss of T-cell inhibition and thus self-tolerance may result in autoantibodies which form immune complexes with either circulating self-antigens or native antigen already within the glomerulus.

However, an alternative and thought-provoking possibility is that autoimmunity may result in excessive activation of the alternative complement cascade. Our biopsy showed moderate C3 staining and only weak IgM staining on immunofluorescence microscopy. It remains classified as immune complex-mediated MPGN based on current diagnostic criteria, given that the intensity of staining of C3 was only one grade level above IgM. Yet, the possibility of complement-mediated MPGN (i.e., C3 glomerulonephritis) must be considered given that IgM may also result from nonspecific trapping, especially when the staining is weak. A recent report by Ashour et al. showed similar C3-dominant subepithelial and mesangial deposits with sequential exposure to pembrolizumab and nivolumab, though in their case it was IgG predominant deposition [16]. Our case hints more to the potential of complement-mediated disease. If checkpoint inhibitors can activate the complement cascade should be a topic of study in the future. We believe our case adds to the growing body of literature that checkpoint inhibitors can cause a wide spectrum of immune-mediated kidney pathology, spanning the gamut of both interstitial and glomerular disease.

We acknowledge that given our patient's bone marrow transplant history and prior mitomycin C exposure, there is potential that the MPGN pattern could be secondary to thrombotic microangiopathy (TMA) from either chronic graft-versus-host disease or drug-induced TMA. However, there were no glomerular or vascular lesions suggestive of active or prior TMA. Moreover, the kidney injury temporally and rapidly occurred after nivolumab initiation with biopsy immunofluorescence showing subendothelial immune deposits, a finding not seen with TMA. The deposits suggest an immune-mediated injury from checkpoint inhibition. Finally, the patient's AKI and nephrotic syndrome resolved upon withdrawal of nivolumab and provision of a prolonged steroid course.

Checkpoint inhibitor-associated AKI is most commonly associated with AIN. Here, we report the first case of MPGN directly due to nivolumab therapy. Given the increasing popularity of cancer immunotherapy, recognition that checkpoint inhibitors may cause glomerular disease is paramount to management of AKI in our oncology patients.
